# Poor patients’ knowledge about venous thromboembolism and its therapy is associated with increased risk of major bleeding and discontinuation of anticoagulation: A cohort study

**DOI:** 10.1097/MD.0000000000038697

**Published:** 2024-07-12

**Authors:** Aleksandra Gołąb, Dariusz Plicner, Małgorzata Konieczyńska, Elżbieta Broniatowska, Anetta Undas

**Affiliations:** a Faculty of Medicine and Dentistry, Pomeranian Medical University in Szczecin, Szczecin, Poland; b Krakow Centre for Medical Research and Technologies, John Paul II Hospital, Krakow, Poland; c Unit of Experimental Cardiology and Cardiac Surgery, Faculty of Medicine and Health Sciences, Andrzej Frycz Modrzewski Krakow University, Krakow, Poland; d Department of Cardiovascular Surgery and Transplantation, John Paul II Hospital, Krakow, Poland; e Department of Diagnostic Medicine, John Paul II Hospital, Krakow, Poland; f Department of Thromboembolic Diseases, Institute of Cardiology, Jagiellonian University Medical College, Krakow, Poland; g Faculty of Medicine and Health Sciences, Andrzej Frycz Modrzewski Krakow University, Krakow, Poland.

**Keywords:** anticoagulation, follow-up, knowledge, major bleeding, venous thromboembolism

## Abstract

It has been shown that patients’ knowledge about venous thromboembolism (VTE) and its therapy is suboptimal, which might reduce compliance and worsen prognosis. We investigated whether low VTE patients’ knowledge affects their clinical outcomes during long-term follow-up. We evaluated 151 consecutive patients (51.8 ± 15.7 years) after unprovoked VTE, who were recruited from the outpatient clinic (Krakow, Poland). All patients received anticoagulant treatment, mostly with direct oral anticoagulants (n = 113, 74.8%). The modified Jessa Atrial fibrillation Knowledge Questionnaire (JAKQ-VTE; 16 questions) was used to assess the knowledge of VTE and anticoagulant therapy. During a median follow-up of 58.0 months, VTE recurrence, major bleeding, and anticoagulation withdrawal were recorded. The median percentage of correct responses was 62.5% (12.5–100%) and was inversely correlated with age (*P* < .01). Diabetic patients and those with positive family history of VTE had lower overall scoring compared to the remainder (both *P* < .05). Major bleeding (n = 10, 6.6%) and anticoagulation withdrawal (n = 28, 18.5%), but not VTE recurrence (n = 12, 7.9%), were associated with lower overall scoring compared to the remainder (48.8% ± 12.5% vs 63.8% ± 16.3%, *P* = .003 and 55.3% ± 14.7% vs 64.4% ± 16.3%, *P* = .040, respectively). Major bleeding was independently associated with the female sex (hazard ratio [HR] 6.18; 95% confidence interval [CI] 1.15–33.19, *P* = .034), younger age (HR per 10 years 0.55; 95% CI 0.34–0.90, *P* = .016), OAC therapy discontinuation (HR 6.69; 95% CI 1.62–27.70), and lower overall scoring of JAKQ-VTE (HR 0.60 per 10 percentage points; 95% CI 0.40–0.92, *P* = .019). Insufficient knowledge about VTE and anticoagulant treatment predisposes to a higher risk of major bleeding and therapy discontinuation, but not VTE recurrence in unprovoked VTE patients during long-term follow-up.

## 1. Introduction

Venous thromboembolism (VTE), that involves both deep vein thrombosis (DVT) and pulmonary embolism (PE), is associated with a long-term risk of recurrence. The highest annualized event rate is observed in the absence of transient or persistent risk factors (7.9% per patient-year vs 1.0% per patient-year for VTE provoked by surgery in the first 12 months after the withdrawal of anticoagulant treatment).^[1,2]^ Well-established predictors of VTE recurrence include: male sex, proximal DVT, concomitant PE, presence of a filter in the vena cava or unprovoked VTE events.^[3–6]^ If anticoagulant therapy is discontinued in patients following unprovoked VTE, the cumulative incidence of recurrence is about 11% at 1 year, 19.6% at 3 years, 29.1% at 5 years, and 39.9% after 10 years.^[7]^ Randomized controlled trials showed that the extended use of oral anticoagulants decreases the risk of recurrent VTE by 80–90%.^[8]^ Although effective thromboprophylaxis and anticoagulant treatment are available, the prediction of recurrent VTE and identification of subjects at the highest risk are still challenging.^[2,9]^

Patients taking direct oral anticoagulants (DOAC) instead of vitamin K antagonists (VKA) is rapidly increasing worldwide. DOAC compared to VKA have similar efficacy, no need of routine coagulation monitoring, no dietary interactions, greater patient convenience, and a significantly lower risk of major bleeding.^[10,11]^ Nevertheless, the education of both the VTE patients and the caregiver about the importance of adherence, bleeding risk, and drug interaction just as proper dosing and its regimen, is recommended and its level is suboptimal worldwide.^[12]^ The RIETE registry showed that a substantial proportion of VTE patients received nonrecommended doses and/or regimens of DOAC, which was related to a 10 times higher risk of VTE recurrence.^[13]^

Despite better safety profile of DOAC compared to VKA, bleeding complications are still the most common adverse events observed on anticoagulation. To estimate the risk of anticoagulation-related bleeding in patients with VTE, several prediction scores have been proposed including the VTE-BLEED score, which was validated both in nonselected VTE patients and in patients with unprovoked VTE, as well as for all available classes of oral anticoagulants.^[14–16]^ Nevertheless, data on the usefulness of proposed scores in the management of patients with unprovoked VTE receiving extended anticoagulation are still limited.^[17,18]^ It is noteworthy that the bleeding risk may be partly affected by failure of a patient to understand the importance of compliance, resulting from insufficient awareness of disease and knowledge about the therapy.^[19]^

The Jessa Atrial fibrillation Knowledge Questionnaire (JAKQ) was developed and validated in 2016 by Desteghe et al,^[20]^ in order to assess knowledge of patients about atrial fibrillation and self-management. We have already demonstrated a prognostic value of insufficient patients’ knowledge about atrial fibrillation and oral anticoagulants, assessed using the JAKQ score.^[21]^ In 2019, we developed a modified Jessa Atrial fibrillation Knowledge Questionnaire adopted for venous thromboembolism (JAKQ-VTE) patients divided into the same 4 sections containing 16 multiple-choice questions and we showed that knowledge of VTE and anticoagulation is unsatisfactory among anticoagulated patients following provoked or unprovoked events.^[22]^

Little is known about the impact of insufficient patients’ knowledge about VTE and oral anticoagulant (OAC) therapy on recurrent VTE or major bleeding during long-term therapy. Therefore, in this cohort study, we sought to assess whether knowledge gaps assessed with the JAKQ-VTE scores can predict clinical outcomes in unprovoked VTE patients with indication for prolonged anticoagulation.

## 2. Materials and methods

### 2.1. Patients

The study was performed in accordance with the Declaration of Helsinki and received approval of the Jagiellonian University Medical College Ethical Committee (approval number 1072.6120.136.2018). All patients gave informed consent to participate in the study.

We studied 151 consecutive patients with prior unprovoked VTE, that is, in the absence of any identifiable risk factors, which were recruited from the outpatient clinic in Krakow (Poland) from November 2016 to June 2018 and represented a subset of the group studied previously.^[22]^ Patients who reported hormonal contraception, pregnancy, childbirth, surgery or trauma, hospitalization or long travel (>4 hours) in the last 3 months, and active cancer were classified as having provoked VTE and were excluded from the final analysis. The diagnosis of DVT was established by a positive finding on color duplex ultrasound (visualization of an intraluminal thrombus in the calf, popliteal, femoral or iliac veins). The diagnosis of PE was based on the presence of characteristic symptoms and positive results of angiography computed tomography. Obesity was diagnosed in subjects with body mass index ≥ 30 kg/m^2^. Type 2 diabetes (T2D) was defined as a history of diabetes, use of hypoglycemic agents or fasting glycemia> 7 mmol/L. The postthrombotic syndrome was defined as chronic venous symptoms and/or signs secondary to DVT using the Villalta scale.^[23]^ Previous myocardial infarction, stroke, liver cirrhosis, chronic kidney disease, and autoimmune diseases were established based on medical records. Positive family history of VTE was defined as a history of documented VTE in at least one first-degree relative. Higher education was defined as a completion of college or university. The state of sex or gender analysis was assessed using the Sex and Gender Equity in Research (SAGER) guidelines.^[24]^

We calculated VTE-BLEED score of each patient as described previously.^[16]^ Patients were divided into 2 groups: patients at a high risk (VTE-BLEED score ≥ 2) and at a low risk (VTE-BLEED score < 2) for major bleeding. Uncontrolled hypertension was defined by a value of the systolic blood pressure of ≥ 140 mm Hg at baseline. Anemia was diagnosed if the value of the hemoglobin was < 13 g/dL for men and < 12 g/dL for women. Renal dysfunction was defined by a value of the creatinine clearance of < 60 mL/min, which was calculated with the Cockcroft–Gault formula. A history of major bleeding was diagnosed if the patient had a history of major bleeding, which consisted of a reduction in the hemoglobin level by at least 2 g/dL, transfusion of at least 2 units of blood, or symptomatic bleeding in a critical area or organ according to the International Society of Thrombosis and Hemostasis guidelines.^[25]^

### 2.2. Questionnaire

The JAKQ-VTE was used to assess VTE patients’ knowledge about their disease and OAC treatment.^[20]^ Briefly, the questionnaire comprised multiple-choice questions (4 answers), which only one was correct answer, including option “I do not know” to avoid guessing. Among 16 questions, 8 were about VTE in general, 5—about the OAC therapy, and 3—about VKA or DOAC treatment, depending on the anticoagulant was used by a given patient. One point was awarded for a correct answer, and 0 for an incorrect or “I do not know” option. Based on answered questions a total score was calculated, and was displayed as the percentage (16 correct responses = 100%). Physicians collected demographic and clinical data on the study participants.

### 2.3. Follow-up

Follow-up was performed from March 2019 through December 2022. All participants were contacted at least twice a year by telephone or via in-person medical visits. Patients were asked about the occurrence of any thrombotic or bleeding event, identifiable risk factors and changes in OAC treatment, in particular OAC discontinuation. The primary endpoint was a composite of symptomatic VTE recurrence and major bleeding, which were also analyzed separately. Recurrent symptomatic VTE was diagnosed using the same criteria as at the index event. Patients suspected for recurrent PE underwent spiral computed tomography, followed by pulmonary angiography in the case of a high clinical probability of PE despite normal computed tomography scans. Recurrent DVT was diagnosed on the basis of suggestive signs or symptoms, that is, enhanced pain, tenderness, edema, and redness, if there was incompressibility of a proximal vein segment previously free from thrombi, or the finding of a more than 4 mm increase of the vein diameter in a previously noncompressible vein segment as compared with the last measurement. Major bleeding was defined as nonsurgical bleeding resulting in blood transfusion (at least 2 units of packed red blood cells) or a significant fall in hemoglobin levels (at least 2 g/dL) or bleeding to critical organs.^[25]^ The investigators who assessed clinical outcomes during follow-up were unaware of the JAKQ-VTE scores.

### 2.4. Statistical analysis

Power calculation was performed using data published in our previous report.^[21]^ Given the probability of *α* error at 0.05, the study was powered to have *β* error of 0.2 of detecting a 25% difference in knowledge level between patients who experienced major bleeding and those free of such complication during follow-up. In order to demonstrate such a difference or greater, 84 patients were required in the group free of major bleeding and 10 in the major bleeding group. Twelve patients are required in the VTE group and 100 patients in the control group to detect the 26% difference in knowledge level between these groups with the *β* error equal to 0.2 and the *α* error of 0.05. The calculations were performed using G* Power Version 3.0.10.^[26]^

Categorical variables were presented as numbers and percentages. Continuous variables were expressed as mean (±standard deviation) or median (interquartile range [IQR]), as appropriate. Normality was assessed by Shapiro–Wilk test. Equality of variances was assessed using Levene test. Differences between groups were compared using the Student or Welch *t* test depending on the equality of variances for normally distributed variables. The Mann–Whitney *U* test was used for nonnormally distributed continuous variables. Nominal variables were compared by either Pearson chi-squared test or Fisher exact test as appropriate. Survival curves for the endpoints were estimated by the Kaplan–Meier method and for their comparison the log-rank test was used. The proportional hazard Cox regression was used to determine potential predictors of studied events. The resulted model was presented as hazard rations (HR) with 95% confidence intervals (CI). The proportional hazard assumption was verified by the Schoenfeld Residuals test. The deviance residuals were applied for assessing the model goodness of fit and the c-statistics (c-index, area under the curve) was used to estimate the predictive accuracy of this model. A *P*-value < .05 was considered statistically significant. All statistical analyses were performed with Statistica 13 software (StatSoft, Tulsa, Oklahoma) and using R, Version 4.1.0 (R Foundation for Statistical Computing, Vienna, Austria).

## 3. Results

### 3.1. Baseline characteristics

We studied 151 patients with a history of unprovoked VTE event, aged 51.8 ± 15.7 years, including 84 (55.6%) men (Table 1). Women were markedly older than men (55.7 ± 14.8 years vs 48.7 ± 15.7 years, *P* = .006) and more often suffered from autoimmune diseases (25.4% vs 3.6%, *P* < .001), whereas history of previous myocardial infarction was noticed more common in men (11.9% vs 3.0%, *P* = .044). There were no other sex-related differences with regard to demographic and clinical variable. The median time between VTE diagnosis and enrollment was 29.0 (IQR 11.0–72.0) months. There were 63 (41.7%) patients with isolated DVT and 31 (20.5%) with isolated PE, while 58 (38.4%) individuals had DVT with concomitant PE. The first unprovoked VTE episode occurred in 86 (57.0%) of the studied patients, and they tended to be younger than those with recurrent VTE event (54.5 ± 15.2 years vs 49.4 ± 15.8 years, *P* = .059). In terms of VTE prevention, the majority of patients (n = 113, 74.8%) were treated with DOAC, including rivaroxaban (60.2%), apixaban (19.5%), and dabigatran (20.3%), whereas 28 patients (18.5%) received VKA therapy. The remaining patients (n = 10, 6.6%) received a low-molecular-weight heparin. According to the VTE-BLEED score, there were 39 (25.8%) patients at high risk of major bleeding, while 112 (74.2%) remaining patients were classified to the low-risk group (Table 2).

**Table 1:** Baseline characteristics of patients in relation to study endpoints.

Variable	All patient (n = 151)	Recurrence of VTE			Major bleeding			Any event		
		No (n = 139)	Yes (n = 12)	*P*-value	No (n = 141)	Yes (n = 10)	*P*-value	No (n = 130)	Yes (n = 21)	*P*-value
Age (yr)	51.8 ± 15.7	52.1 ± 15.4	48.2 ± 18.8	.41	52.0 ± 16.0	48.6 ± 10.4	.51	52.3 ± 15.7	48.3 ± 15.6	.28
Male, n (%)	84 (55.6)	76 (54.7)	8 (66.7)	.42	81 (57.4)	3 (30.0)	.11	73 (56.2)	11 (52.4)	.75
Higher education, n (%)	49 (32.5)	48 (34.5)	1 (8.3)	.10	47 (33.3)	2 (20.0)	.38	46 (35.4)	3 (14.3)	.08
Comorbidities, n (%)										
Varicose veins	77 (51.0)	70 (50.4)	7 (58.3)	.60	73 (51.8)	4 (40.0)	.53	67 (51.5)	10 (47.6)	.74
Obesity	60 (39.7)	54 (38.8)	6 (50.0)	.54	55 (39.0)	5 (50.0)	.52	50 (38.5)	10 (47.6)	.43
History of VTE	36 (23.8)	31 (22.3)	5 (41.7)	.13	32 (21.2)	4 (40.0)	.21	28 (21.5)	8 (38.1)	.10
Postthrombotic syndrome	31 (20.5)	27 (19.4)	4 (33.3)	.27	29 (20.6)	2 (20.0)	.99	25 (19.2)	6 (28.6)	.38
Chronic kidney disease	30 (20.0)	29 (20.9)	1 (8.3)	.29	29 (20.6)	1 (10.0)	.42	29 (22.3)	1 (7.1)	.08
T2D	27 (17.9)	22 (15.8)	5 (41.7)	**.041**	24 (17.0)	3 (30.0)	.38	19 (14.6)	8 (38.1)	**.026**
Current smoking	25 (16.6)	22 (15.8)	3 (25.0)	.41	22 (20.6)	3 (30.0)	.24	19 (14.6)	6 (28.6)	.11
Autoimmune disease	20 (13.2)	18 (12.9)	2 (16.7)	.66	18 (12.8)	2 (20.0)	.62	17 (13.1)	3 (14.3)	.99
Previous MI	12 (7.9)	10 (7.2)	2 (16.7)	.24	12 (8.5)	0 (0.0)	.99	10 (7.7)	2 (9.5)	.67
Previous stroke	9 (6.0)	9 (6.5)	0 (0.0)	.99	8 (5.7)	1 (10.0)	.47	8 (6.2)	1 (4.8)	.99
Liver cirrhosis	4 (2.6)	4 (2.9)	0 (0.0)	.55	3 (2.1)	1 (10.0)	.13	3 (2.3)	1 (7.1)	.52
OAC treatment, n (%)										
VKA	28 (18.5)	26 (18.7)	2 (16.7)	.99	25 (17.7)	3 (30.0)	.39	23 (17.7)	5 (23.8)	.55
DOAC	113 (74.8)	104 (74.8)	9 (75.0)	.96	107 (75.9)	6 (60.0)	.27	99 (76.2)	14 (66.7)	.35
Rivaroxaban	68 (60.2)	64 (61.5)	4 (44.4)	.48	64 (59.8)	4 (66.7)	.99	61 (61.6)	7 (50.0)	.40
Apixaban	22 (19.5)	21 (20.2)	1 (11.1)	.99	22 (20.6)	0 (0)	.59	21 (21.2)	1 (7.1)	.30
Dabigatran	23 (20.3)	19 (18.3)	4 (44.4)	.08	21 (19.6)	2 (33.3)	.60	17 (17.2)	6 (42.8)	**.036**
VTE characteristics, n (%)										
DVT isolated	63 (41.7)	58 (41.7)	5 (41.7)	.99	59 (41.8)	4 (40.0)	.99	54 (41.5)	9 (42.9)	.91
PE isolated	31 (20.5)	30 (21.6)	1 (8.3)	.46	29 (20.6)	2 (20.0)	.99	29 (22.3)	2 (9.5)	.25
DVT with concomitant PE	58 (38.4)	52 (37.4)	6 (50.0)	.54	54 (38.3)	4 (40.0)	.99	48 (36.9)	10 (47.6)	.35
VTE recurrence	65 (42.8)	61 (43.9)	6 (50.0)	.68	63 (44.7)	4 (40.0)	.99	57 (43.8)	10 (47.6)	.75
The time since VTE diagnosis (mo)	29.0 (11.0–72.0)	29.0 (11.0–6.0)	27.5 (13.3–75.0)	.81	33.0 (11.0–72.0)	19.0 (8.3–30.0)	.40	32 (11.3–70.0)	21.0 (8.0–72.0)	.73
The time since initiating the current OAC therapy (m)	11.0 (6.0–24.0)	12.0 (7.0–24.0)	9.0 (3.8–19.0)	.34	12.0 (7.0–24.0)	11.0 (5.3–22.8)	.72	12.0 (7.0–24.0)	10.0 (5.0–19.0)	.24

Values are shown as mean ± standard deviation, number (%) or median (interquartile range). Bold values denote differences with *P* < .05.

DOAC = direct oral anticoagulant, DVT = deep vein thrombosis, GI = gastrointestinal, MI = myocardial infarction, OAC = oral anticoagulant, PE = pulmonary embolism, VKA = vitamin K antagonists, VTE = venous thromboembolism.

**Table 2:** Prevalence of the VTE-BLEED score items and percentage of the JAKQ-VTE score according to risk of bleeding in the study population.

Variable	VTE-BLEED low risk (n = 112)	VTE-BLEED high risk (n = 39)	All (n = 151)
Male with uncontrolled arterial hypotension, n (%)	10 (8.9)	6 (15.4)	16 (10.6)
Anemia, n (%)	2 (1.8)	17 (43.6)	19 (12.6)
History of bleeding, n (%)	1 (0.9)	5 (12.8)	6 (4.0)
Age ≥ 60 yr, n (%)	17 (15.2)	35 (89.7)	52 (34.4)
Renal dysfunction, n (%)	2 (1.8)	28 (71.8)	30 (19.9)
Active cancer, n (%)	0	0	0
JAKQ-VTE score (%)	68.8 (56.3–75.0)	56.3 (50.0–68.8)^a^	62.5 (50.0–75.0)

Values are shown as number (%) or median (interquartile range).

JAKQ-VTE = Jessa Atrial fibrillation Knowledge Questionnaire adopted for venous thromboembolism.

a *P* = .033 for difference between the VTE-BLEED low-risk and VTE-BLEED high-risk groups.

**Figure 1.**
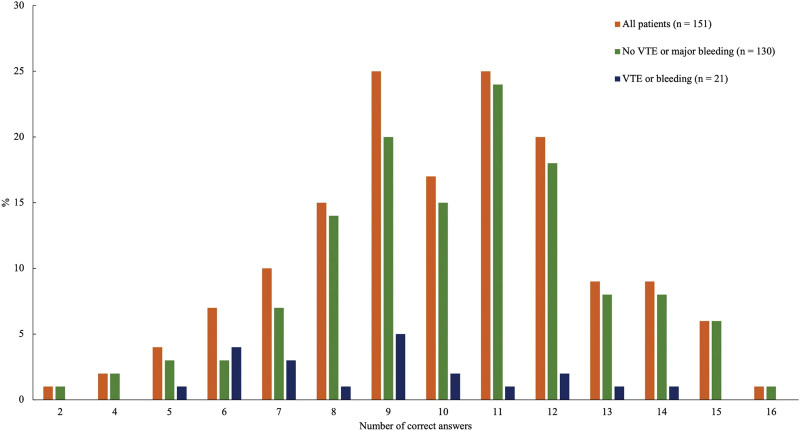
Distribution of correct responses (%) to 16 questions. VTE = venous thromboembolism.

The median percentage of correct responses was 62.5% (IQR 50.0–75.0; minimum 12.5% and maximum 100%; Fig. 1) and was inversely correlated with age (*r* = −0.28, *P* < .01). Patients who had better knowledge about VTE in general, had higher scoring while replying to the questions about OAC therapy (*r* = 0.25, *P* = .002). Men tended to be more likely to provide correct answers as compared to women (65.0% ± 16.3% vs 60.0% ± 16.3%, *P* = .054, respectively). Diabetic patients and those with positive family history of VTE had lower overall scoring compared to the remainder (55.6% ± 13.1% vs 64.4% ± 16.9%, *P* = .008, and 56.5% ± 15.9% vs 65.9% ± 15.8%, *P* = .007, respectively). Moreover, higher risk of bleeding according to the VTE-BLEED score was related to lower baseline overall scoring (Table 2). Patients with other comorbidities as well as those with higher education did not differ in their knowledge compared to the remainder. Of note, there were no differences in overall scoring between patients taking DOAC and those on VKA (63.1% ± 15.6% vs 66.3% ± 12.5%, *P* = .31, respectively). However, patients on apixaban had higher overall scoring compared to those on rivaroxaban or dabigatran (75.0% [57.8–85.9%] vs 62.5% [50.0–68.8%], *P* = .030 and 62.5% [43.8–68.8%], *P* = .034, respectively), with no difference between the results for patients treated with rivaroxaban and dabigatran (*P* = .87).

### 3.2. Anticoagulant therapy during follow-up

The overall median of the anticoagulant treatment since VTE diagnosis was 88.0 months (IQR 70.0–125.5 months). During a median follow-up of 58.0 (IQR 55.0–60.0) months, all of the anticoagulated patients were on DOAC therapy (n = 129, 85.4%) as a secondary VTE prevention, including 73 (56.6%) taking rivaroxaban, 41 (31.8%) apixaban, and 15 (11.6%) dabigatran. Twenty-seven of the 28 patients who received VKA at baseline, were switched to DOAC treatment (n = 17 [63.5%] to rivaroxaban, n = 7 [25.9%] to apixaban, and n = 3 [11.1%] to dabigatran).

There were 28 (18.5%) patients who discontinued OAC treatment, including 22 (14.6%) patients, who after a median follow-up of 34.0 (IQR 22.3–42.8) months interrupted anticoagulant treatment and 6 (3.9%) patients, who were switched to DOAC therapy, and after a median of 12.0 (IQR 9.0–19.0) months, they stopped taking these drugs. Patients who discontinued OAC therapy were characterized by a higher prevalence of a positive VTE family history and those receiving dabigatran (39.3% vs 20.3%, *P* = .034, and 32.1% vs 11.4%, *P* = .013, respectively). Moreover, patients who stopped OAC treatment had a shorter time between VTE diagnosis and recruitment, and thereby shorter treatment duration compared to the remainder (18.5 [IQR 5.8–38.5] months vs 33.0 [IQR 12.0–72.0] months, *P* = .022, and 8.5 [IQR 3.0–18.3] months vs 12.0 [IQR 7.0–24.0] months, *P* = .037, respectively).

Patients who stopped OAC treatment had lower baseline overall scoring (56.3% ± 15.6% vs 64.4% ± 16.3%, *P* = .040). Analysis of all 16 questions showed lower scores while replying to 3 specific questions. Patients who stopped OAC therapy had poor knowledge about the definition of PE (25.0% vs 74.0%, *P* < .001), possible thrombotic consequences of DVT (35.7% vs 84.6%, *P* < .001) and its diagnosis (39.3% vs 60.2%, *P* = .041) compared to the remainder.

### 3.3. Recurrent VTE during follow-up

We documented 12 (7.9%) recurrent VTE events (1.7 per 100 patient-years), including 6 isolated DVT and 6 DVT with concomitant PE. Recurrent VTE events occurred after a median of 38.3 (IQR 31.8–49.0) months since enrollment. There were no differences in baseline demographic and clinical variables in patients with and without VTE recurrence, except for a more common diagnosis of T2D in the former group (*P* = .041, Table 1). As expected, patients with VTE recurrence stopped anticoagulant therapy more commonly (75.0% vs 13.7%, *P* < .001).

**Figure 2.**
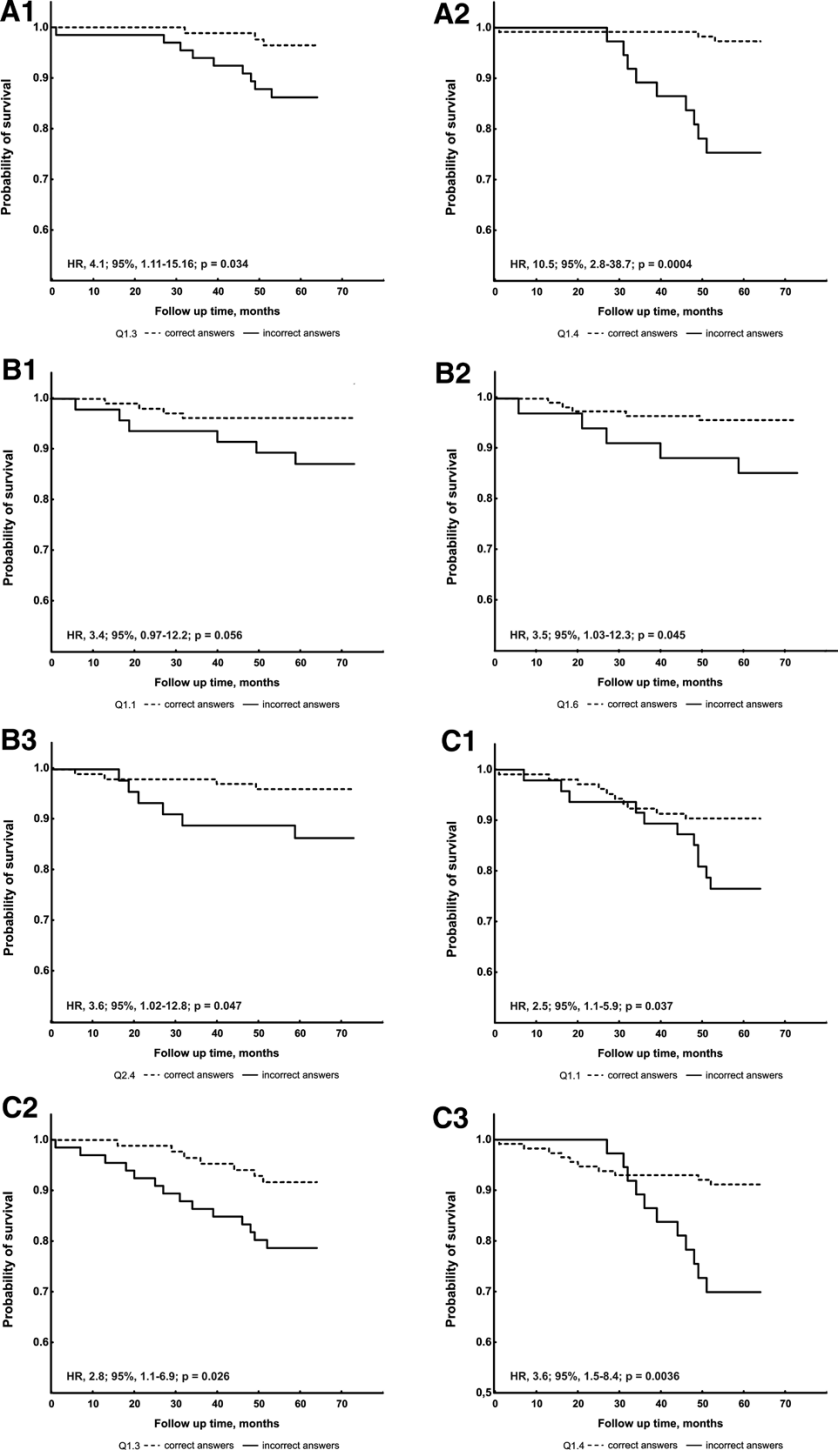
Kaplan–Meier curves in patients with history of unprovoked venous thromboembolism (VTE). The recurrent VTE (A), major bleeding (B) and any event (C) rates in patients who answered correctly (A1, Q1.3; A2, Q1.4; B1, Q1.1; B2, Q1.6; B3, 2.4; C1, Q1.1; C2, Q1.3; C3, Q1.4) compared to those who provided a wrong answer. HR = hazard ratio, Q = question, VTE = venous thromboembolism.

There was no difference in overall scoring between patients with VTE recurrence and the remainder (*P* = .385). However, the former subgroup was less likely to know about DVT diagnosis (25.0% vs 54.3%, *P* = .023) and the association of DVT with PE (25.0% vs 73.5%, *P* < .001, Table 3). Those patients, who answered wrong to these questions, had a markedly higher risk of VTE recurrence (HR 4.10; 95% CI 1.11–15.16, *P* = .034, and HR 10.46; 95% CI 2.83–38.69, *P* < .001, respectively; Fig. 2 panel A1 and A2).

**Table 3:** Specific topics addressed in the JAKQ-VTE with the percentage of correct responses among venous thromboembolism patients in relation to recurrence, major bleeding, and composite endpoint.

Parameter	All patients (n = 151)	No recurrent VTE (n = 139)	Recurrent VTE (n = 12)	*P*-value	No bleeding (n = 141)	Major bleeding (n = 10)	*P*-value	No study endpoint (n = 130)	Study endpoint (n = 21)	*P*-value
Questions about VTE in general										
Q.1.1 PE occurs when pulmonary arteries are partially occluded, most often by clots	103 (68.2)	98 (64.9)	6 (50.0)	.19	100 (70.9)	4 (40.0)	.07	94 (72.3)	10 (47.6)	**.023**
Q1.2 VTE is not always accompanied by pain or edema of the limb	56 (37.1)	52 (34.4)	4 (33.3)	.99	54 (38.3)	2 (20.0)	.32	50 (34.5)	6 (28.6)	.38
Q1.3 Venous ultrasound should be performed to detect DVT	85 (56.3)	82 (54.3)	3 (25.0)	**.023**	81 (57.4)	4 (40.0)	.33	78 (60.0)	7 (33.3)	**.022**
Q1.4 DVT can lead to PE	114 (75.5)	111 (73.5)	3 (25.0)	**<.001**	106 (75.2)	8 (80.0)	.99	104 (80.0)	10 (47.6)	**.001**
Q1.5 Blood thinners prevent the recurrence of the disease	53 (35.1)	48 (31.8)	5 (41.7)	.75	48 (34.0)	5 (50.0)	.32	44 (33.8)	9 (42.9)	.42
Q1.6 Travel by air or car for more than 6–8 h increases the risk of VTE	117 (77.5)	108 (71.5)	9 (75.0)	.73	112 (79.4)	5 (50.0)	**.046**	104 (80.0)	13 (61.9)	.09
Q1.7 Being overweight increases the risk of VTE	117 (77.5)	108 (71.5)	9 (75.0)	.73	111 (78.2)	6 (60.0)	.23	103 (79.2)	14 (66.7)	.26
Q1.8 Blood thinners should be used always, for at least 3 mo after diagnosis of VTE	26 (17.2)	25 (16.6)	1 (8.3)	.69	26 (18.4)	0 (0.0)	.21	25 (19.2)	1 (4.8)	.13
Questions about OAC therapy										
Q2.1 Patients with VTE should always take their blood thinners, especially if VTE is unprovoked	118 (78.1)	108 (71.5)	10 (83.3)	.99	110 (78.0)	8 (80.0)	.99	101 (77.7)	17 (81.0)	.99
Q2.2 Possible side effects of blood thinners are the occurrence of bleedings and longer bleeding times in case of injuries	134 (88.7)	124 (82.1)	10 (83.3)	.63	127 (90.1)	7 (70.0)	.09	117 (90.0)	17 (81.0)	.26
Q2.3 VTE patients may only take painkillers based on paracetamol	77 (51.0)	72 (47.7)	5 (41.7)	.50	73 (51.8)	4 (40.0)	.53	68 (52.3)	9 (42.9)	.42
Q2.4 When VTE patients regularly have minor nose bleeds (that spontaneously cease) they should contact the general practitioner or specialist, while continuing to take their blood thinners	103 (68.2)	95 (62.9)	8 (66.7)	.99	99 (70.2)	4 (40.0)	**.07**	91 (70.0)	12 (57.1)	.24
Q2.5 If a VTE patient needs an operation, he/she should consult a doctor to discuss possible options	94 (62.3)	85 (56.3)	9 (75.0)	.54	90 (63.8)	4 (40.0)	.17	81 (62.3)	13 (61.9)	.83
Questions about VKA										
Q3.1 VTE patients taking VKA should have their blood thinning checked at least once a month	61 (40.4)	55 (36.4)	6 (50.0)	.23	57 (40.4)	4 (40.0)	.99	51 (39.2)	10 (47.6)	.62
Q3.2 When VTE patients taking VKA have forgotten to take their blood thinner they should still take their forgotten pill (immediately or at the next dose)	22 (14.6)	19 (12.6)	3 (25.0)	.69	21 (14.9)	1 (10.0)	.99	18 (13.8)	4 (19.0)	.99
Q3.3 INR is a measure to check how thick or how thin the blood is	58 (38.4)	52 (34.4)	6 (50.0)	.65	56 (49.7)	2 (20.0)	.17	50 (38.5)	8 (38.1)	.23
Questions about DOAC										
Q4.1 For patients taking DOAC, it is important to take their blood thinner at the same time every day	110 (72.8)	100 (66.2)	10 (83.3)	.99	103 (73.0)	7 (70.0)	.51	94 (72.3)	16 (76.2)	.64
Q4.2 When VTE patients taking DOAC have forgotten to take their blood thinner, they can still take that dose, unless the time till the next dose is less than the time after the missed dose	72 (47.7)	66 (43.7)	6 (50.0)	.75	69 (48.9)	3 (30.0)	.26	63 (48.5)	9 (42.9)	.32
Q4.3 The DOAC card should be shown to their general practitioner and specialist by VTE patients	15 (9.9)	12 (8.6)	4 (33.3)	**.008**	15 (10.6)	0 (0.0)	.07	12 (9.2)	3 (14.3)	.47

Values are shown numbers (%). Bold values denote differences with *P* < .05.

DOAC = direct oral anticoagulant, DVT = deep vein thrombosis, INR = international normalized, JAKQ-VTE = Jessa Atrial fibrillation Knowledge Questionnaire adopted for venous thromboembolism, OAC = oral anticoagulant, PE = pulmonary embolism, VKA = vitamin K antagonists, VTE = venous thromboembolism.

Discontinuation of OAC therapy and T2D were associated with VTE recurrence (*P* < .001 and *P* = .041, respectively). The multiple Cox proportional hazards model showed that younger age, T2D and OAC treatment discontinuation were independent predictors for recurrent VTE (Table 4).

**Table 4:** Multiple Cox proportional hazard models for prediction of study endpoints.

Variable	VTE recurrence		Major bleeding		Any event	
	HR (95% CI)	*P*-value	HR (95% CI)	*P*-value	HR (95% CI)	*P*-value
Age per 10 yr	0.630 (0.914–0.999)	**.044**	0.551 (0.339–0.895)	**.016**	0.588 (0.429–0.805)	**<.001**
Female sex	1.242 (0.297–5.185)	.767	6.176 (1.149–33.188)	**.034**	2.452 (0.815–7.378)	.111
T2D	9.072 (2.034–40.461)	**.004**	2.057 (0.440–9.625)	.360	5.805 (1.907–17.670)	**.002**
Percentage of correct answers in JAKQ-VTE per 10%	1.028 (0.655–1.614)	.904	0.604 (0.397–0.919)	**.019**	0.791 (0.576–1.087)	.149
OAC therapy discontinuation	19.134 (4.658–78.604)	**<.001**	6.691 (1.616–27.700)	**.009**	12.315 (4.471–33.922)	**<.001**

C statistic: 0.87, 0.87 and 0.85, respectively. Bold values denote statistical significance at the *P* < .05 level.

HR = hazard ratio, JAKQ = Jessa AF Knowledge Questionnaire, OAC = oral anticoagulant, T2D = type 2 diabetes, VTE = venous thromboembolism.

### 3.4. Major bleeding during follow-up

There were 10 (6.6%) major bleeding events (1.4 per 100 patient-years), including 4 (2.6%) gastrointestinal hemorrhages (Table 5). The median time from enrollment to the event was 22.5 (IQR 16.5–34.3) months. There were no differences in demographic and clinical variables in patients with and without major bleeding (Table 1). The patients who bled stopped OAC treatment as a result of such complications a more common compared to those free of this complication (n = 5 [50.0%] vs n = 23 [16.3%], *P* = .020). Another 5 patients who experienced major bleeding were switched from VKA/DOAC to DOAC therapy (n = 3 to apixaban and n = 2 to rivaroxaban). Major bleeding led to discontinuation of OAC therapy as reflected by higher proportion of this adverse event among patients who stopped OAC therapy compared to the remainder (17.9% vs 4.1%, *P* = .008).

**Table 5:** Major bleedings in patients on anticoagulation during follow-up.

Bleeding, n = 10	OAC therapy	Dosage (mg)	JAKQ-VTE total score in %
GI bleeding, n = 4	Rivaroxaban	15	31.3
	Rivaroxaban	20	43.8
	Dabigatran	2 × 150	43.8
	Dabigatran	2 × 150	56.3
Menorrhagia, n = 3	Rivaroxaban	20	75.0
	Warfarin	3	37.5
	Rivaroxaban	20	37.5
Posttraumatic ICH, n = 1	Rivaroxaban	20	56.3
Extraperitoneal bleeding, n = 1	Apixaban	2 × 2.5	50.0
Hematuria, n = 1	Rivaroxaban	20	56.3

GI = gastrointestinal bleeding, ICH = intracerebral hemorrhage, JAKQ = Jessa Atrial Fibrillation Knowledge Questionnaire, OAC = oral anticoagulant, VTE = venous thromboembolism.

Patients who experienced major bleeding had lower overall scoring compared to the remainder (46.9% [37.5–56.3%] vs 62.5% [56.3–75.0%], *P* = .003). Three of the 4 gastrointestinal bleeding patients and 2 of 3 patients with history of menorrhagia had baseline scoring below the median value of 46.9% (Table 5). As shown in Table 3, they tended to be less aware of the relation between long travels and increased risk of VTE (50.0% vs 79.4%, *P* = .046) and appropriate management in case of minor nose bleeds that spontaneously ceased (40.0% vs 70.2%, *P* = .07). Incorrect responses to these 2 questions were associated with a 3.5-fold higher risk of major bleeds (HR 3.55; 95% CI 1.03–12.28, *P* = .045, and HR 3.60; 95% CI 1.02–12.78, *P* = .047, respectively; Fig. 2 panel B2 and B3). Moreover, patients who experienced major bleeding tended to be more likely to provide wrong answer to the question about the definition of PE (40.0% vs 70.9%, *P* = .07). Kaplan–Meier curves showed a tendency to higher event rate of major bleeding in those subjects (*P* = .056, Fig. 2 panel B1).

As expected, discontinuation of OAC therapy was associated with major bleeding events (*P* = .020). As shown in Table 4, major bleeding was independently associated with the female sex, younger age, OAC therapy discontinuation, and lower overall scoring of JAKQ-VTE.

### 3.5. Combined study endpoint

The primary study endpoint occurred in 21 (13.9%) patients (2.9 per 100 patient-years). The median time interval since enrollment to the index event was 33.0 (IQR 21.3–47.5) months. We documented 5 (3.3%) deaths and none of them was related to recurrent VTE or major bleeding. Patients who experienced recurrent VTE or major bleeding more commonly suffered from T2D (Table 1). Moreover, they discontinued OAC treatment more frequently compared to the remainder (61.9% vs 11.5%, *P* < .001). One patient, who discontinued OAC therapy due to major bleeding at 18.0 months, experienced recurrent VTE after 25.0 months from anticoagulation withdrawal.

Patients who experienced recurrent VTE or major bleeding had lower baseline overall scoring, which was associated with the primary outcome (56.3% [43.8–62.5%] vs 65.6% [56.3–75.0%], *P* = .014). Subjects with any event had a lower proportion of correct responses to the questions about the definition of PE (47.6% vs 72.3%, *P* = .023), possible thrombotic consequences of DVT (33.3% vs 60.0%, *P* = .022) and its diagnosis (47.6% vs 80.0%, *P* = .001, Table 3). We observed increased risk of any event in patients, who provided wrong responses to the above questions (HR 2.49; 95% CI 1.06–5.86, *P* = .037, and HR 3.58; 95% CI 1.52–8.44, *P* = .004, and HR 2.80; 95% CI 1.13–6.94, *P* = .026, respectively; Fig. 2 panel C1, C3 and C2).

Anticoagulant therapy withdrawal and T2D were related to the combined study endpoint (*P* < .001 and *P* = .026, respectively). Multiple Cox proportional hazards model showed that younger age, T2D, and OAC treatment discontinuation were independent risk factors of the combined endpoint (Table 4).

## 4. Discussion

To our knowledge, this study is the first to show that poor knowledge assessed using the JAKQ-VTE is associated with worse clinical outcomes, particularly a higher risk of major bleeding, in anticoagulated patients following VTE, which occurred in the absence of transient or persistent risk factors. We found no association of insufficient knowledge with VTE recurrence during almost 5 years of follow-up. From a practical point of view, we identified out of 16 questions in the JAKQ-VTE a few specific issues of key importance for the prediction of recurrent VTE and/or major bleeding in patients with unprovoked VTE. Our findings highlight the practical value of the questionnaire tested in unprovoked VTE patients to help identify those at risk for adverse events. This study supports educational efforts to improve patients’ knowledge largely for their safety on long-term OAC therapy, which is relevant given the current guidelines encouraging prolonged therapy following VTE in a growing subset of VTE patients.^[27,28]^

The present study extends the previous report, which demonstrated that the JAKQ-VTE could be a user-friendly assessment tool in daily practice.^[22]^ We found that low total scoring at baseline has a predictive value and it was associated with bleeding, but not with VTE recurrence, which might imply that this approach could be helpful in particular among VTE patients at high bleeding risk. Analysis of all 16 questions showed that patients who experienced major bleeding during follow-up, compared to those free of such complication, tended to be more likely to provide wrong answers to the question about the definition of PE and were less aware of increased risk of VTE during travel by air or car for more than 6–8 hours. Besides worse awareness about the essence of VTE, patients who bled were less likely to know that they should contact the general practitioner or specialist in case of regularly minor nose bleeds, and continuing OAC treatment. These identified knowledge gaps were associated with a significantly increased risk of major bleeding. We did not observe intergroup differences in the correct responses to the question about preferred safe painkiller, which patient may use on OAC treatment, however, this knowledge was insufficient in both groups. Our findings suggest that educational efforts should be focused on the awareness of safe OAC therapy, which may reduce the risk of adverse outcomes in the long-term follow-up.

Given the growing evidence for the value of the VTE-BLEED score,^[14–17]^ the current report provides clinically relevant data. In our cohort, the major contribution to the high bleeding risk according to the VTE-BLEED score was advanced age, renal dysfunction, and anemia. There were no subjects with cancer, because we focused on unprovoked VTE population. It is noteworthy that unprovoked VTE patients who were classified to the high bleeding risk group had lower baseline level of knowledge about the disease and anticoagulation, reflected by the JAKQ-VTE result, compared to those low risk of this adverse event. Our findings underscore the need of grater educational efforts aimed the high bleeding risk group, in which the benefits from better education could be easier to observe. Thus, further studies are needed to establish the role of both tools in a daily medical practice.

It might be speculated that the lack of understanding of the essence of the disease may be the reason for worse patient compliance and finally results in OAC treatment withdrawal.^[19]^ Despite the fact that there was no difference in the overall scoring between patients with VTE recurrence and those free of this complication, we identified 2 specific questions on the JAKQ-VTE, which were related with increased risk of VTE recurrence. Those patients were less likely to know about possible thrombotic consequences of DVT, including PE, and the diagnostic method of DVT diagnosis. Importantly, in this study we identified poor knowledge as a factor increasing the risk of therapy discontinuation among VTE patients, which is associated with increased VTE recurrence.^[8,29]^

Our cohort is representative in terms of unprovoked VTE real-life patients. We followed patients for a median of 58.0 months and 0.7% on-treatment of recurrent VTE just as much major bleeding were diagnosed in the first year. The rates observed by us were similar to or slightly lower than those reported in randomized DOAC trials during 12 months of drug administration in patients who had completed 3 to 12 months of initial OAC treatment and had indications for its extension.^[30–32]^ A multicenter cohort study by Bortoluzzi et al, who evaluated 450 unprovoked VTE patients on rivaroxaban (more than 60% patients received longer than 2 years of treatment, and around 20% completed the 5-year therapy), showed that the incidence rate of VTE recurrence was 1.7 per 100 person–years (95% CI, 0.9–3.1), which is with line in our findings (1.7 per 100 person–years [95% CI, 0.9–2.9]). The frequency of major bleeding during follow-up in that study was also similar to the current rates observed by us at 2 years (2.9% vs 3.3%).^[33]^

We failed to show any impact of overall scoring on VTE recurrence, which was unexpected. This might be partly explained by a relatively small group size and the occurrence of additional factors affecting the risk of recurrence. We identified T2D as a predictor of VTE recurrence among unprovoked VTE patients, which is in line with some previous reports including a meta-analysis by Bai et al^[34]^ who observed that this comorbidity increased the risk of first and subsequent VTE events. The exact mechanism underlying behind this phenomenon is still unclear, but it has been speculated that hypercoagulability, along with impaired fibrinolysis observed in patients with T2D, might contribute to this association.^[34]^ Recently we have reported that T2D is associated with a lower major bleeding acceptance assessed using the Bleeding Ratio, that is, a maximum number of major bleeds that people were willing to endure to prevent one similar recurrent VTE episode.^[35]^ However, the current report showed that insufficient knowledge about VTE and OAC use, but not T2D occurrence, was a predictor of bleeding recorded during follow-up.

Interestingly, the current study showed that during follow-up older patients had lower risk of any event compared with younger subjects, despite the observed negative correlation between age and percentage of correct answers in JAKQ-VTE. It has been reported that advancing age is associated with a higher incidence of first VTE event and bleedings related to the OAC treatment.^[11]^ However, data regarding the influence of aging on its recurrence in unprovoked VTE patients on long-term anticoagulation are limited and yielded inconsistent results.^[29,33,36]^ Some authors have reported increased risk,^[36]^ and others found no relationship with aging.^[29,33]^ Moreover, Speed et al showed that in VTE patients on rivaroxaban the strongest predictor of the good adherence to this medication was older age (adjusted odds ratio per 10 years 1.21; 95% CI 1.06–1.39), while the frequency of VTE recurrence was low, at just 0.6% over 2 years.^[19]^ These discrepancies between the results of the former studies and the current report can be partly explained by differences in patient characteristics and study design. Nevertheless, this issue requires further studies.

Data on how to improve knowledge level and increase the compliance to prophylaxis and treatment for VTE patients are insufficient. The supplementary education over and above what most patients receive from health care providers in the usual course of anticoagulant prescribing is suggested by the 2018 American Society of Hematology (ASH) guideline, however, this recommendation is based on very low certainty in the evidence about effects.^[12]^ Simultaneously, the 2018 ASH guideline suggests against using electronic reminders or a visual medication schedule to improve adherence to anticoagulation, due to large uncertainty regarding net health benefit/harm by using these methods. In the study by Tran et al^[37]^ higher patients’ knowledge about OAC treatment, female sex, and no history of VTE were associated with better adherence to anticoagulation among patients with indications to their use. It has been observed that higher medication concerns, for example, about possible side effects, might be the reason for the worse compliance with recommendations.^[18]^ Thus, our findings advise that targeted education in form of discussion with the patients the results of JAKQ-VTE may improve their knowledge as well as adherence to anticoagulant-medication regimens in VTE patients, and thus may decrease the risk of adverse events. Major studies in this area could establish the role of these efforts on prognosis in the subset of unprovoked VTE patients on extended OAC treatment.

Our study has several limitations. Firstly, the size of the study group was relatively small, however, representative for patients with history of VTE, which occurred without transient or persistent risk factors. We did not analyze the impact of other drugs used in the study population, such as statins or aspirin, on clinical outcomes, though statins were shown to lower the risk of first and recurrent VTE.^[38]^ Moreover, asymptomatic VTE events could have been omitted during follow-up, therefore recurrence of VTE may be underestimated in this study. Hereditary thrombophilia was not routinely evaluated as risk factor for VTE in the present study.^[39]^ Finally, we recorded major bleeding, while clinically relevant non-major and minor bleed events were not analyzed.

In conclusion, this study showed that insufficient patients’ knowledge, assessed by the JAKQ-VTE score, can predict the risk of clinical outcomes, but not VTE recurrence, in patients with a history of VTE, which occurred without any identifiable risk factors. Educational efforts improving knowledge about the essence of VTE and its secondary prevention in those patients, who have indications to long-term anticoagulation are needed. It remains to be established the influence of these efforts on prognosis among anticoagulated patients after unprovoked VTE.

## Author contributions

**Conceptualization:** Dariusz Plicner, Małgorzata Konieczyńska, Anetta Undas.

**Data curation:** Małgorzata Konieczyńska, Anetta Undas.

**Formal analysis:** Aleksandra Gołąb, Elżbieta Broniatowska.

**Investigation:** Aleksandra Gołąb, Dariusz Plicner, Małgorzata Konieczyńska, Elżbieta Broniatowska, Anetta Undas.

**Methodology:** Aleksandra Gołąb, Dariusz Plicner, Anetta Undas.

**Supervision:** Dariusz Plicner, Małgorzata Konieczyńska, Elżbieta Broniatowska, Anetta Undas.

**Writing – original draft:** Aleksandra Gołąb, Dariusz Plicner, Anetta Undas.

**Writing – review & editing:** Aleksandra Gołąb, Dariusz Plicner, Małgorzata Konieczyńska, Elżbieta Broniatowska, Anetta Undas.
